# Comparing texture and digestion properties between white and brown rice of indica cultivars preferred by Chinese consumers

**DOI:** 10.1038/s41598-021-98681-7

**Published:** 2021-09-24

**Authors:** Min Huang, Xing Li, Liqin Hu, Zhengwu Xiao, Jiana Chen, Fangbo Cao

**Affiliations:** Crop and Environment Research Center for Human Health, Key Laboratory of Ministry of Education for Crop Physiology and Molecular Biology, Hunan Agricultural University, Changsha, 410128 China

**Keywords:** Biochemistry, Plant sciences, Health care

## Abstract

The consumption of good tasting rice, mainly soft-textured white rice with low amylose content, has substantially increased in China as living standards improve. However, this diet change may increase the risk of developing type II diabetes because the soft-textured white rice is generally less resistant to digestion and has a higher glycemic index. In contrast, intake of brown rice is inversely associated with type II diabetes risk. This study was conducted to test the possibility that brown rice processed from soft-textured cultivars has both acceptable texture and improved health benefits. Texture and digestion properties were compared between white and brown rice of five indica cultivars preferred by Chinese consumers. Mean hardness was 33% higher while mean springiness was 5% lower for cooked brown rice than for cooked white rice. As compared to cooked white rice, cooked brown rice had a 41% longer mean active digestion duration but 31% lower mean glucose production rate and 11% lower mean total glucose production from starch digestion. However, the differences in texture and starch digestion properties between cooked brown and white rice were affected by cultivar identity. Brown rice processed from suitable cultivars with both a relatively thinner bran layer and relatively higher grain amylose content met consumer requirements in terms of acceptable texture and improved health benefits.

## Introduction

China has the largest population of rice consumers in the world, with more than 65% of the population (~ 900 million people) eating rice as a staple food^1^. China has been self-sufficient in rice supply due to an increase in grain yield per unit area by more than 50% since 1980^2^. The sufficient supply of rice has led to dietary changes such as replacing coarse cereals (i.e., cereal grains other than rice and wheat) and whole grains (e.g., brown rice—the rice from which only the husk has been removed) with polished rice (i.e., white rice—the rice from which the husk, bran, and germ have been removed)^3,4^. More recently, as living standards improve in China, the production and consumption of good tasting rice, mainly soft-textured white rice with low amylose content, has substantially increased^5,6^.

There is ample evidence that consuming more white rice is associated with an increased risk of type II diabetes in Asian populations including the Chinese^7,8^. The increased consumption of low amylose, soft-textured white rice may increase the risk of developing type II diabetes because this type of rice is generally less resistant to digestion and has a higher glycemic index^9^. On the contrary, intake of coarse cereals and whole grains including brown rice is inversely associated with type II diabetes risk^10,11^. In addition, whole grain foods have other health benefits, such as reducing the risk of cardiovascular disease and cancer^12,13^, which are of increasing concern both nationally and globally due to their high mortality rates^14^.

By comprehensively considering consumer preferences for soft-textured rice and the health benefits of brown rice, we speculated that brown rice processed from soft-textured cultivars may have both acceptable texture and improved health benefits. To test this, we conducted a study comparing the texture and digestion properties of white and brown rice of five indica cultivars preferred by consumers in China.

## Results

Averaged across five cultivars, the milling recovery rate was 21% higher for brown than for white rice (Table 1). Brown rice had 9% lower mean amylose content compared to white rice. Mean protein content in brown rice was 13% higher than that in white rice. The differences in amylose and protein contents between brown and white rice were affected by cultivar identity. Amylose contents in brown rice of Meixiangzhan 2, Nongxiang 42, Taoyouxiangzhan, and Yuzhenxiang were higher than that in white rice of Xiangyaxiangzhen. Protein contents in brown rice of Meixiangzhan 2, Nongxiang 42, Xiangyaxiangzhen, and Yuzhenxiang were lower than that in white rice of Taoyouxiangzhan.

**Table 1:** Milling recovery rate and amylose and protein content of white and brown rice of five cultivars.

Rice type	Cultivar	Milling recovery rate (g kg^–1^)	Amylose content (mg g^–1^)	Protein content (mg g^–1^)
White rice	Meixiangzhan 2	681	187	59.3
	Nongxiang 42	659	189	53.4
	Taoyouxiangzhan	680	178	72.9
	Xiangyaxiangzhen	570	112	59.0
	Yuzhenxiang	637	179	62.2
	Mean	645 b	169 a	61.4 b
Brown rice	Meixiangzhan 2	775	171	67.2
	Nongxiang 42	794	172	65.2
	Taoyouxiangzhan	808	167	78.5
	Xiangyaxiangzhen	726	101	69.9
	Yuzhenxiang	792	161	67.2
	Mean	779 a	154 b	69.6 a

Means within a column sharing different letters are significantly different from each other at the 0.05 probability level.

Cooked brown rice had 33% higher mean hardness than cooked white rice (Table 2). Mean springiness was 5% lower for cooked brown than white rice, while there were no significant differences in mean cohesiveness and resilience. There was no significant difference in mean chewiness between cooked brown and white rice. The differences between cooked brown and white rice in texture profiles, especially hardness, were influenced by cultivar identity. The hardness values of cooked brown rice of Meixiangzhan 2, Nongxiang 42, Taoyouxiangzhan, and Xiangyaiangzhen were lower than that of cooked white rice of Yuzhenxiang.

**Table 2:** Texture profiles of cooked white and brown rice of five cultivars.

Rice type	Cultivar	Hardness (g)	Springiness	Cohesiveness	Resilience	Chewiness (g)
White rice	Meixiangzhan 2	729	0.769	0.603	0.439	342
	Nongxiang 42	769	0.793	0.612	0.428	366
	Taoyouxiangzhan	753	0.774	0.600	0.395	364
	Xiangyaxiangzhen	603	0.756	0.491	0.341	227
	Yuzhenxiang	992	0.786	0.541	0.393	444
	Mean	769 b	0.776 a	0.569 a	0.399 a	349 a
Brown rice	Meixiangzhan 2	956	0.752	0.550	0.398	352
	Nongxiang 42	938	0.754	0.549	0.388	361
	Taoyouxiangzhan	938	0.725	0.520	0.361	343
	Xiangyaxiangzhen	866	0.723	0.515	0.385	297
	Yuzhenxiang	1415	0.752	0.563	0.418	551
	Mean	1023 a	0.741 b	0.539 a	0.390 a	381 a

Means within a column sharing different letters are significantly different from each other at the 0.05 probability level.

Mean active digestion duration for cooked brown rice was 41% longer than that for cooked white rice (Table 3). The mean glucose production rate was 31% lower for cooked brown rice than for cooked white rice. Cooked brown rice had 11% lower mean total glucose production compared to cooked white rice. Differences in starch digestion properties between cooked brown and white rice were affected by cultivar identity. In particular, cooked brown rice of Xiangyaxiangzhen had a shorter active digestion duration and higher glucose production rate than cooked white rice of Meixiangzhan 2, Nongxiang 42, Taoyouxiangzhan, and Yuzhenxiang.

**Table 3:** Starch digestion properties of cooked white and brown rice of five cultivars.

Rice type	Cultivar	Active digestion duration (min)	Glucose production rate (mg g^–1^ min^–1^)	Total glucose production (mg g^–1^)
White rice	Meixiangzhan 2	88	4.22	373
	Nongxiang 42	100	3.65	366
	Taoyouxiangzhan	86	4.02	344
	Xiangyaxiangzhen	76	4.82	367
	Yuzhenxiang	79	4.33	340
	Mean	86 b	4.21 a	358 a
Brown rice	Meixiangzhan 2	154	2.27	345
	Nongxiang 42	142	2.40	302
	Taoyouxiangzhan	117	2.73	296
	Xiangyaxiangzhen	76	4.35	328
	Yuzhenxiang	115	2.76	317
	Mean	121 a	2.90 b	318 b

Means within a column sharing different letters are significantly different from each other at the 0.05 probability level.

## Discussion

Overall, shifting from white to brown rice resulted in decreased eating quality (i.e., increased hardness and decreased springiness) of cooked rice of tested cultivars. The decreased eating quality of cooked brown rice compared to cooked white rice was mainly attributable to the retained fiber-rich bran layer^15^, which could be reflected by the different milling recovery rates of brown and white rice (Table 1). Protein is abundant in the bran layer of brown rice^15^, and an increase in grain protein content generally leads to a harder gel consistency and a lower paste viscosity of rice flour and decreased eating quality of cooked rice^16,17^. Therefore, in the present study, the decreased eating quality of cooked brown rice compared to cooked white rice was also attributable to an increase in grain protein content.

Grain amylose content is also a key determinant of the eating quality of cooked rice, and higher grain amylose content usually leads to a harder texture of cooked rice^18^. However, in this study, the harder texture of cooked brown rice compared to white rice was not explained by the difference in grain amylose content, because brown rice had lower grain amylose content compared to white rice.

The starch digestion of cooked rice was generally slower due to shifting from white to brown rice. This could explain why intake of brown rice is inversely associated with the risk of developing type II diabetes^10,11^, since rice starch with higher resistance to digestion has a lower glycemic index^19–21^. Grain amylose content is a critical component determining the starch digestion rate of cooked rice, and higher grain amylose content is always associated with a higher resistant starch content and a lower starch digestion rate^9^. However, this was not responsible for the slower starch digestion rate in cooked brown compared to white rice in this study, because the grain amylose content was lower in brown than in white rice.

The starch digestion rate of cooked rice also depends on non-starch components such as fiber, protein, lipids, and polyphenols, which are rich in the bran layer of brown rice^15^. These non-starch components may complex with starch and inhibit starch digestion by affecting starch properties; producing a protective layer around starch; or having antagonistic effects on digestive enzymes^22^. For example, protein present in endosperm can decrease the starch digestion rate of cooked rice by restricting swelling and reducing the surface area of the starch granules^23,24^. Therefore, in this study, the slower starch digestion of cooked rice due to shifting from white rice to brown rice was mainly attributable to the retained bran layer that is rich in non-starch components such as protein.

In addition, this study showed that the differences in texture and starch digestion properties between cooked brown and white rice were affected by cultivar identity. In particular, cooked brown rice of Meixiangzhan 2, Nongxiang 42, and Taoyouxiangzhan had lower hardness and chewiness than cooked white rice of Yuzhenxiang and a slower starch digestion rate than cooked white rice of all cultivars. This outcome indicates that selecting suitable cultivars such as Meixiangzhan 2, Nongxiang 42, and Taoyouxiangzhan and processing their grains into brown rice is expected to provide acceptable texture and benefit health. Based on grain quality traits, Meixiangzhan 2, Nongxiang 42, and Taoyouxiangzhan had a relatively thinner bran layer (i.e., lower difference in milling recovery rate between brown and white rice) and relatively higher grain amylose content among the five tested cultivars (Table 1). This finding suggests that bran layer thickness and grain amylose content are important traits for selecting suitable cultivars to produce tasty and healthy brown rice, and highlights the need for determining the optimum range of these two traits through further investigations with more rice cultivars.

There are some limitations that should be acknowledged. This study evaluated the texture acceptability and health benefits of brown rice only by comparing its physicochemical properties with those of white rice, but not by direct consumer involvement. In this regard, it is advocated that the consumer involvement can play a vital role in food and health research^25,26^. Moreover, there are other grain quality traits, such as aroma and appearance, that determine consumer preference for rice^27^. Therefore, more studies including consumer organoleptic tests and clinical trials are required to comprehensively evaluate the acceptability and feasibility of brown rice processed from soft-textured cultivars.

## Conclusions

Shifting from white to brown rice retains the bran layer with abundant non-starch components such as protein, and consequently leads to decreased eating quality (i.e., increased hardness and decreased springiness) but an increase in health quality (i.e., slower starch digestion) of indica cultivars preferred by Chinese consumers. However, brown rice with acceptable texture and improved health benefits can be obtained by selecting suitable cultivars (e.g., Meixiangzhan 2, Nongxiang 42, and Taoyouxiangzhan) that have a relatively thinner bran layer and relatively higher grain amylose content.

## Methods

Rice grains of five indica cultivars—Meixiangzhan 2, Nongxiang 42, Taoyouxiangzhan, Xiangyaxiangzhen, and Yuzhenxiang—were collected from the research base of the Hengyang Academy of Agricultural Sciences (26° 59′ N, 112° 23′ E), Meihua Village, Hunan Province, China in 2020. These rice cultivars have good taste and a soft texture. In particular, four of the five cultivars (i.e., Meixiangzhan 2, Nongxiang 42, Taoyouxiangzhan, and Yuzhenxiang) won the Gold Award of the Eating Quality Evaluation Contest of High-Quality Indica Rice in China. The use of plants in the present study complies with international, national and/or institutional guidelines.

Rice grain samples were air-dried and then stored for three months before analysis. For each cultivar, 100 g of rice grain samples were de-hulled to obtain brown rice and then half of the brown rice was polished to white rice, using a laboratory-scale milling machine (JGMJ8098, Shanghai Jiading Cereals and Oils Instrument Co., Ltd., Shanghai, China) and closely following the procedure of the International Standard ISO 6646:2011^28^. Milling recovery rates of brown and white rice were calculated by separately dividing the brown and white rice weight by the grain weight.

About 5 g of brown and white rice flours (filtered through 100 mesh) were prepared for each sample to determine amylose content and protein content according to procedures described by Huang et al.^29^. In brief, amylose content was measured with the iodine colorimetric method. The protein content was determined by multiplying N content by a conversion factor of 5.95; N content was measured with an automatic Kjeldahl analyzer (Kjeltec-8400, FOSS, Copenhagen, Denmark).

Approximately 10 g of white and brown rice were soaked in 16 ml of distilled water in an aluminum cup for 30 min, and the aluminum cup was then covered with a lid and placed in an electric rice cooker (GDF-2003; Zhuhai Gree Group Co., Ltd., Zhuhai, China) containing approximately 700 ml of boiling water and allowed to steam for 40 min. The lid of the electric rice cooker remained locked for 20 min after steaming was complete. Texture profiles (hardness, springiness, cohesiveness, resilience, and chewiness) of the cooked rice were determined using a texture analyzer (Rapid TA^+^; Shanghai Tengba Instrument Technology Co. Ltd., Shanghai, China).

Starch digestion properties of the cooked rice were determined using an in vitro method. In detail, 100 mg samples of cooked rice were subjected to in vitro digestion to determine the amount of glucose produced per unit fresh weight at six digestion times (15, 60, 120, 180, 240, and 300 min) using a Glycemic Index Analyser (NutriScan GI20; Next Instruments, Condell Park, NSW, Australia). The starch digestion process of the cooked rice (i.e., the change in the amount of glucose produced over time) was fitted to an exponential association model, y = a[1 − EXP(− bx)], based on goodness of fit and biological plausibility (CurveExpert 1.4; Hyams Development, Chattanooga, TN, USA). The digestion parameters of the cooked rice, including active digestion duration, total glucose production, and glucose production rate, were estimated with y at 95% of a (0.95a) using the following formulas: active digestion duration = LN(0.05)/− b; total glucose production = 0.95a; and glucose production rate = total glucose production/active digestion duration; a and b were obtained from the fitting.

All data were compared between brown and white rice by paired *t*-tests (DPS 18.10; Analytical Software, Hangzhou, China). Statistical significance was set at the 0.05 probability level.

## Data Availability

All data generated or analysed during this study are included in the article.
